# Case report: Thoracic vertebral abscess caused by Salmonella via diagnosed next-generation sequencing

**DOI:** 10.3389/fmed.2024.1419356

**Published:** 2024-08-16

**Authors:** Xiao-guang Cao, Jun-xi Ni, Chong-jian Huang

**Affiliations:** ^1^Department of Emergency Medical Center, The First Affiliated Hospital of University of Science and Technology of China (Anhui Provincial Hospital), Hefei, Anhui, China; ^2^The Third People's Hospital of Hefei, Hefei, Anhui, China; ^3^Emergency Department, Suzhou Hospital of Anhui Medical University (Suzhou Municipal Hospital of Anhui Province), Suhou, Anhui, China

**Keywords:** thoracic vertebra, *Salmonella Typhi*, NGS, infection, diagnosis

## Abstract

The genus Salmonella consists of Gram-negative bacteria with various serotypes. It commonly causes bacterial infections that affect the intestines. Infection can occur in humans and animals through the ingestion of contaminated food or water, or through contact with infected animals or environments. Complications commonly include intestinal hemorrhage and perforation, though vertebral osteomyelitis is rarely observed. Therefore, in patients with spinal cord abscesses, The genus Salmonella is typically not considered a likely pathogen, especially in the absence of typical symptoms. In this case, the limited information provided by traditional cultivation methods, particularly under the influence of antibiotics. However, next-generation sequencing (NGS) unexpectedly detected Salmonella, which assisted in formulating the final treatment plan. This underscores the role and clinical value of NGS in pathogen identification.

## Case presentation

### Clinical part

**Figure 1:**
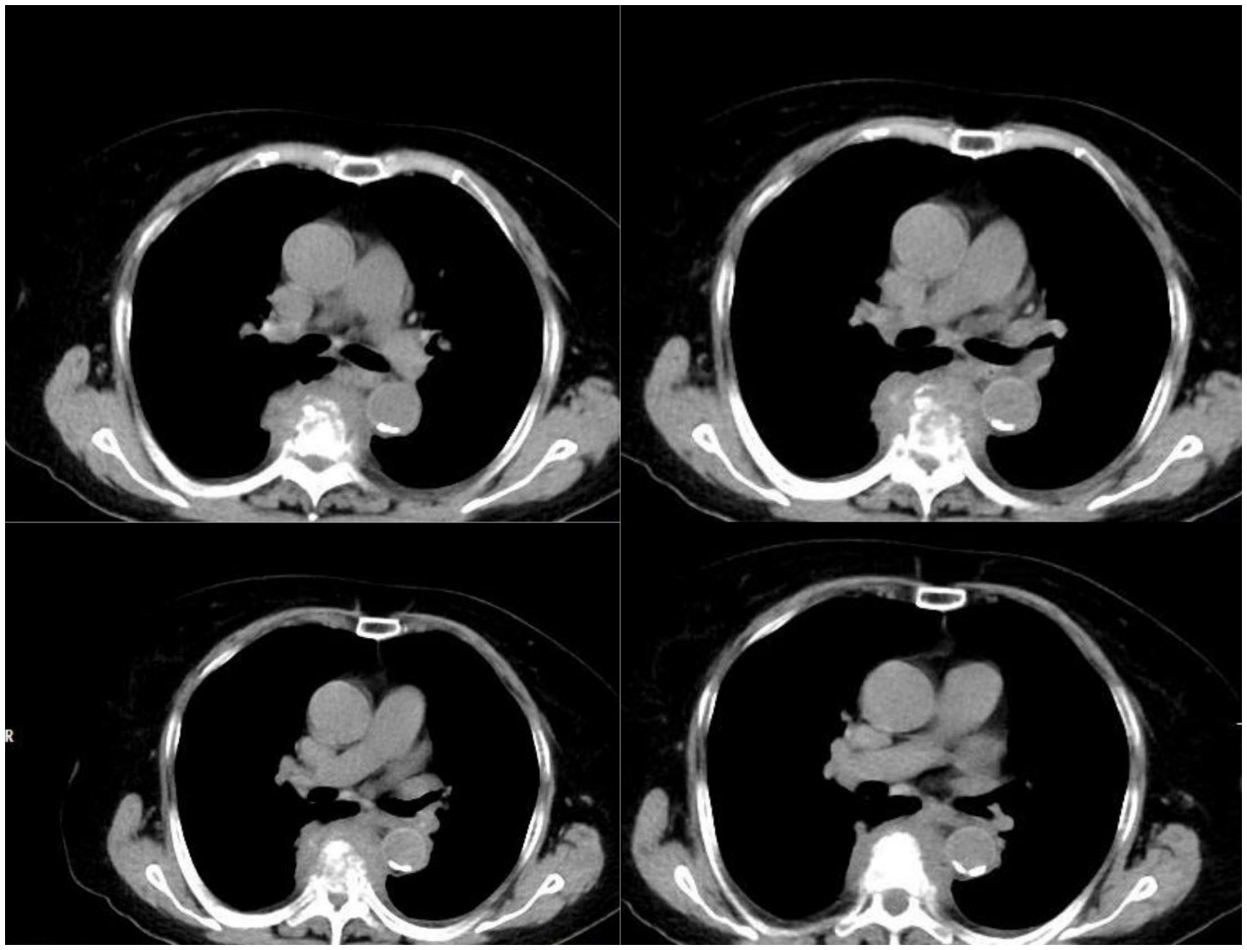
Compression fractures of T6 and T7 vertebrae with associated surrounding soft tissue mass.

A 76-year-old female patient presented with chest and back pain accompanied by fever, approximately 38.2°C, about 1 month prior to hospital admission, with normal limb mobility at that time. The local hospital initially diagnosed her with gallstones and cholecystitis, and she underwent cholecystectomy and received anti-infective treatment. Post-surgery, her temperature improved, but the pain persisted. She revisited the local hospital due to unrelieved pain, where she was considered to have a gastric ulcer and was treated with proton pump inhibitors among other medications, yet without symptom improvement. Two days before presenting to our hospital for further treatment, the patient experienced a gradual decrease in bilateral lower limb strength. During her stay, she had intermittent fever, peaking around 38.2°C, with no significant gastrointestinal symptoms. Emergency CT indicated a thoracic vertebral fracture at T6/T7 (Figure 1), suspected to be caused by vertebral infection. Subsequently, the patient was admitted to the orthopedics department. Physical examination upon admission revealed normal development, good nutritional status, natural facial expression, and moderate body build. The patient was alert and cooperative during the examination, with primary positive findings including loss of pain and touch sensation below the level of the xiphoid process, grade 0 strength in both lower limbs, positive bilateral Babinski sign, loss of saddle area touch sensation, and absence of the anal reflex. Admission tests showed a white blood cell count of 15.41×10^9/L, monocytes at 0.881×10^9/L, lymphocytes at 2.01×10^9/L, neutrophils at 12.971×10^9/L, C-reactive protein at 131.8 mg/L, and a temperature of 37.4°C. Considering the patient’s test results and local epidemiological investigation, the diagnosis was considered to be an infection caused by *Mycobacterium tuberculosis*, and a treatment regimen primarily consisting of anti-tuberculosis drugs (levofloxacin, isoniazid, amikacin, rifampicin, ethionamide, and pyrazinamide) was initiated. Concurrently, related surgical examinations were completed, and on the third day of admission, surgery was performed (posterior spinal canal decompression with internal fixation by pedicle screw and lateral bone grafting), along with the placement of a wound drainage tube, and the excised tissue was sent for culture. Based on intraoperative findings, the doctor suspected a *Staphylococcus aureus* infection, and the anti-infective treatment regimen was adjusted to cefoperazone-sulbactam plus vancomycin.

### NGS results and final treatment plan

**Figure 2:**
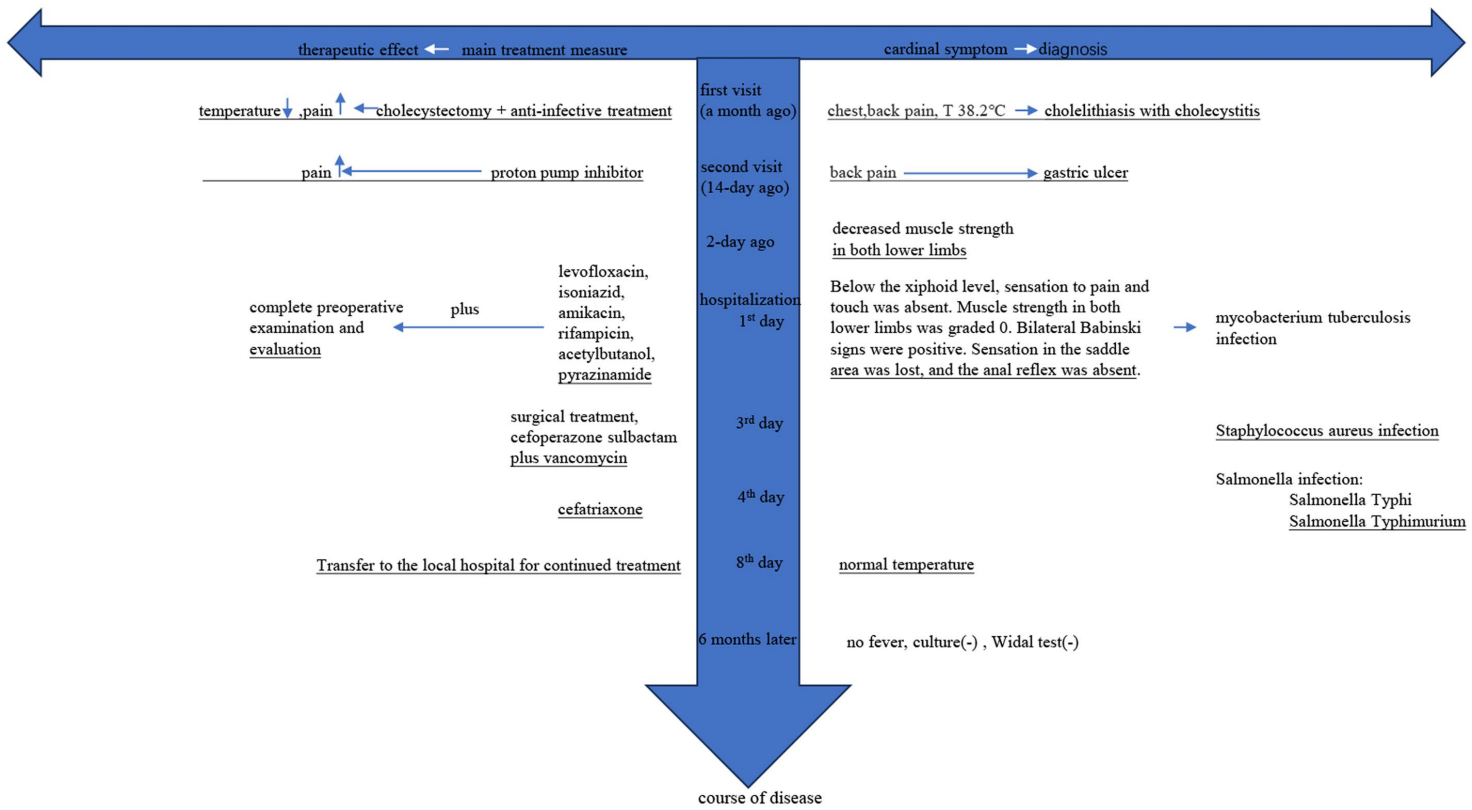
“
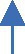
” indicates no improvement in symptoms; “
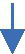
” indicates improvement in symptoms; (−) indicates negative result.

On the fourth day of admission, 65 reads were identified as *Salmonella enterica* serovar Typhimurium and 19 reads were identified as *Salmonella enterica* serovar Typhi, and this result was also supported by the polymerase chain reaction (PCR) verification. Based on the results of domestic epidemiological investigations, the anti-infection treatment plan was adjusted to ceftriaxone (1). The patient’s temperature and inflammatory markers showed a trend of improvement, and she was subsequently transferred to a local hospital for continued treatment. Approximately 6 months later, follow-up revealed no fever, and stool culture and agglutination tests were negative, and no clinical sign of recurrence was observed in the spine (Figure 2).

## Discussion

Spinal infections are serious clinical conditions that can lead to severe consequences. *Mycobacterium tuberculosis* and *Staphylococcus aureus* are typically considered the most likely causative pathogens (2). Salmonella infections are not uncommon in China and include various serovars, such as *Salmonella Typhi*, *Salmonella Typhi*murium, and Salmonella Uzaramo (3–5). These infections are usually transmitted via the fecal-oral route and primarily cause gastrointestinal diseases (6). However, Salmonella-induced spondylitis is very rare in clinical practice. Similar cases have been reported, but most lesions occur in the lumbar spine (7) and cervical spine (8).

Due to the similar imaging characteristics observed in X-ray, CT, and MRI scans, Salmonella-induced spondylitis can easily be misdiagnosed as a tumor, Guillain-Barré syndrome, atypical Lyme disease, atypical pneumonia, scrub typhus, or tuberculosis (9). Currently, Each commonly used clinical testing method has its own advantages and disadvantages (10). The primary diagnostic methods for infections caused by these pathogens include bacterial culture of the affected tissue, blood culture, and the Widal test (11). Culture is considered the gold standard; however, it is prone to various influencing factors such as contamination, antibiotic use, and the collection of specimens from infected areas. The Widal test is a commonly utilized clinical testing method for typhoid fever, which relies significantly on the presence of typical symptoms for its application. Clinicians are required to first consider the diagnosis of typhoid fever before proceeding with this test.

For the initial diagnosis of spinal cord infections, early detection primarily depends on medical history, clinical symptoms, epidemiological data, and imaging studies (11–13). For junior doctors, this complexity may result in missed diagnoses or misdiagnoses (14). Culture remains the gold standard. However, due to the challenges associated with collecting specimens from spinal cord infections, unlike sputum and blood samples, these cannot be easily repeated. Currently, there is no universally accepted antigen, molecular, or nucleic acid-based detection method available that could expedite pathogen identification for patients and physicians. The absence of rapid and reliable detection techniques makes the diagnosis of spinal cord infections particularly difficult, and delays in diagnosis and inappropriate treatment can result in permanent damage (15). Therefore, clinicians are in need of a simple and effective method for pathogen isolation and detection. While culture-based methods continue to be the standard for diagnosing the cause of infections, they are hampered by long processing times and low sensitivity in practical applications. Although molecular and serological detection techniques based on the PCR have been developed to identify pathogens, their utility is restricted to detecting known pathogens that are included in the test panel or suspected pathogens identified by medical professionals. In this case, it was necessary to use NGS to identify Salmonella before designing primers for confirmation, which indirectly highlights the limitations of PCR.

Currently, genomic detection methods are widely used in pathogen detection (3, 5, 6). As a clinically mature technology, NGS can detect thousands of pathogens in a single test and further analyze their subtypes. Consequently, it can decrease the number of required tests and the time needed for diagnosis, eliminate the need for repeated specimen collection, and refine the scope of detection. The detected pathogens can be classified as either pathogenic or opportunistic. Opportunistic pathogens cause disease in humans when the host’s immune system is compromised or when they colonize an unusual site. During the interpretation process, further analysis is conducted based on the patient’s condition and the type of specimen collected to enhance sensitivity and specificity. Thus, NGS can serve as a supportive tool for diagnosing spinal cord infections, providing reliable evidence for complex and clinically challenging cases (16). This approach also introduces several additional potential advantages. During the treatment process of the patient, an initial consideration was given to *Mycobacterium tuberculosis* infection, based on epidemiological investigations and medical history. Following surgery, a bacterial infection was suspected; however, these suspicions were ultimately determined to be misdiagnoses. In this scenario, NGS was utilized to detect pathogens that traditional diagnostic methods could not effectively identify. Ultimately, NGS played a crucial role in the accurate identification of the causative pathogens, enabling the development of appropriate treatment plans (1). Meanwhile, this method offers extra benefits, with a detection cycle of only 12–24 h, which is crucial for optimizing antimicrobial treatment and reducing the use of anti-infective medications.

## Data availability statement

The original contributions presented in this research are included in the paper. The raw sequence data reported in this paper have been deposited in the Genome Sequence Archive in National Genomics Data Center, China National Center for Bioinformation/Beijing Institute of Genomics, Chinese Academy of Sciences, under accession number subCRA028354 that are publicly accessible at https://ngdc.cncb.ac.cn/gsa, and further inquiries can be directed to the corresponding author. For the further inquiries, please direct your questions to the corresponding author.

## Ethics statement

The studies involving humans were approved by Ethics Committee of the First Affiliated Hospital of the University of Science and Technology of China (Anhui Provincial Hospital). The studies were conducted in accordance with the local legislation and institutional requirements. Written informed consent for participation was not required from the participants or the participants’ legal guardians/next of kin in accordance with the national legislation and institutional requirements. Written informed consent has been obtained from the patient for the publication of any potentially identifiable information contained within this article.

## Author contributions

X-gC: Writing – original draft, Writing – review & editing. J-xN: Writing – original draft, Writing – review & editing. C-jH: Conceptualization, Validation, Writing – original draft, Writing – review & editing.
