# Supplementary figures and images for: Case report: Thoracic vertebral abscess caused by Salmonella via diagnosed next-generation sequencing
Source: Front Med (Lausanne). 2024 Aug 16;11:1419356. doi: 10.3389/fmed.2024.1419356 (PMC11362080; doi:10.3389/fmed.2024.1419356)

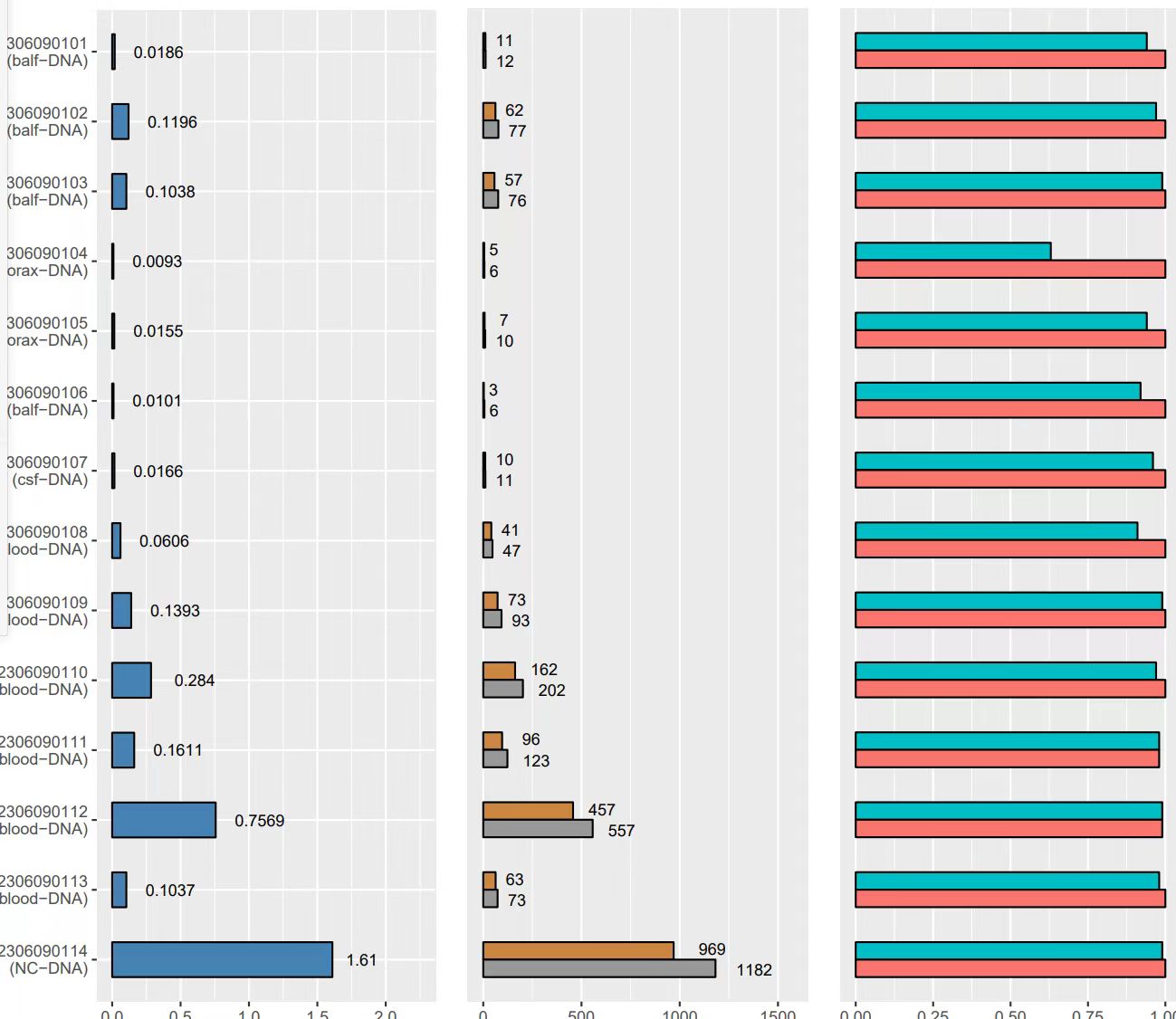


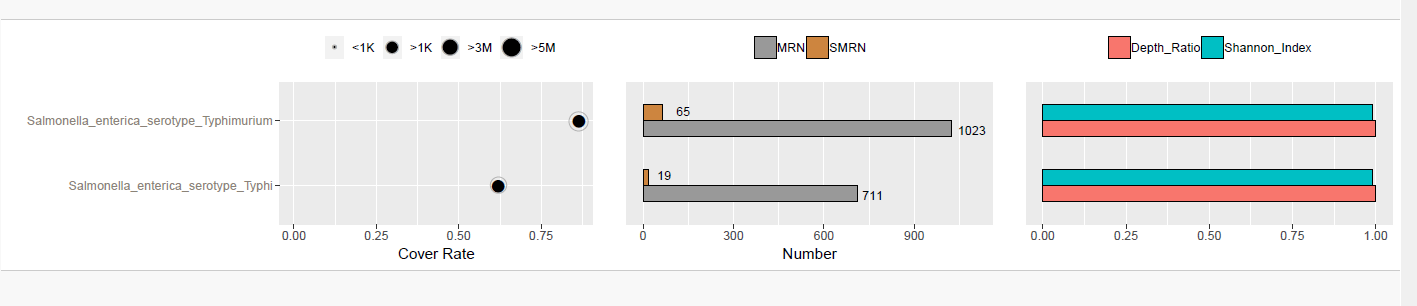


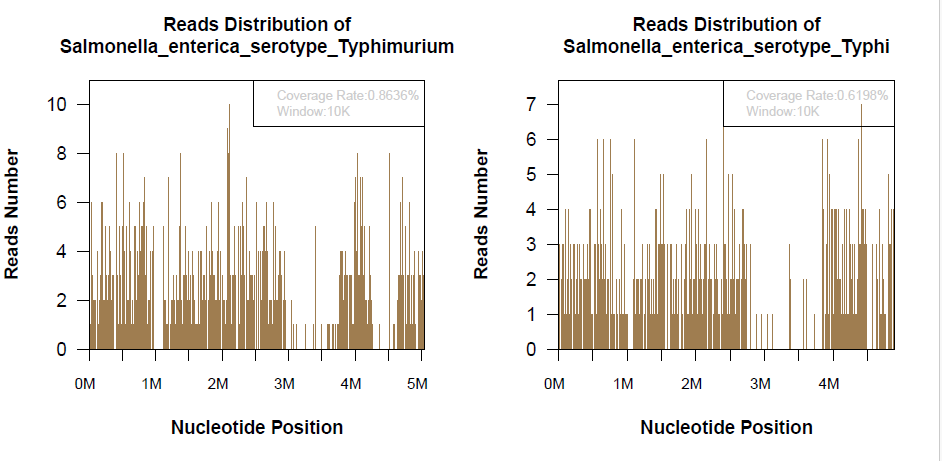

Supplement: Supplementary file 1 [file Data_Sheet_1.docx]
