# Supplementary material for: Case report: Thoracic vertebral abscess caused by Salmonella via diagnosed next-generation sequencing
Source: Front Med (Lausanne). 2024 Aug 16;11:1419356. doi: 10.3389/fmed.2024.1419356 (PMC11362080; doi:10.3389/fmed.2024.1419356)
Supplement: Supplementary file 2 [file Data_Sheet_2.docx]

**DNA Extraction and Fragmentation:** The extracted DNA was first fragmented into pieces of 100-300 bp. Subsequently, the DNA was subjected to end-repair at both the 3' and 5' ends, resulting in blunt-end DNA.

**Library Construction:** Blunt-end DNA was then ligated with barcodes, completing the library construction. The library DNA was subsequently amplified to increase concentration. The reagents used were from the PMseq™ High-Throughput Pathogen Detection Kit, which includes end-repair reagents, adapter ligation reagents, PCR reaction mixture, PCR primers, and barcode reagents.

**Sequencing Data Processing:** The FASTQ (FQ) data generated after sequencing underwent the following processing steps:

1. Adapter trimming
2. Filtering of low-quality sequences
3. Filtering of low-complexity sequences
4. Filtering of excessively short sequences
5. Filtering of redundant sequences
6. Removal of human sequences
7. Sequence alignment
8. Species annotation

Data processing was performed using kraken2.1.2 and bwa0.7.17-r1188. Sequence alignment was conducted against the BGI comprehensive database, which includes the NCBI NT database, covering 17,500 microbial sequences.
